# Collectanea Medica, Consisting of Anecdotes, Facts, Extracts, Illustrations, Queries, Suggestions, &c.

**Published:** 1818-12

**Authors:** 


### COLLECTANEA MEDICA,

CONSISTING OF

### ANECDOTES, FACTS, EXTRACTS, ILLUSTRATIONS, QUERIES, SUGGESTIONS, &c.

RELATING TO THE

*History or the Art of Medicine, and the Auxiliary Sciences.*

Quicquid agunt medici,
nostri farrago libelli.

### *On Mental Alienation.* (From the *Dictionaire des Sciences Medicales.*)

MENTAL ALIENATION is a generic term, intended to express the common character of the different species of aberrations of reason; the vast picture of which is presented to our view in an extensive hospital, when arranged with order and precision.

Observation has so often manifested a sort of concatenation between certain predispositions to mental alienation and the appearance of it on the concurrence of certain events, that it may often be anticipated with tolerable certainty. Thus, certain singularities and eccentricities of conduct, and particularly when similar to those of some other member of the same family who may have been insane, and a peculiar vividness of sentiment, announce the danger of mental derangement, especially when they appear at certain periods of life. Analogous effects may be produced by physical causes, as—injuries of the head from a blow or fall, excessive intemperance, the consequences of an acute or chronic disease, sudden suppression of hæmorrhage, and the repulsion of a cutaneous eruption. Some of the above causes are rare, others frequent: in many instances, they are enveloped in obscurity from family considerations, want of attention, or studied concealment; but, in comparing together the numerous particular histories of mental alienation of our own and other nations, which have been related in collections of cases, or noticed in the records of public establishments, more general and less equivocal results may be obtained.

What an example of confusion and disorder is an assemblage of insane persons, when distributed without method, and observed in a superficial and indiscriminate manner; but, with continued attention, and an accurate investigation of the symptoms proper to each, they may be classed in a general manner, and particularly distinguished by the fundamental lesions of the understanding and will, having previously made an abstraction of their innumerable varieties. Delirium, more or less marked, is on all occasions connected, in many insane persons, with a state of agitation or fury, more or less violent: this has generally been designated by the term *mania.* Delirium may be exclusive, and confined to a particular series of objects, with a sort of inertness, accompanied with acute and concentrated affections: this has been called *melancholy.* Occasionally the passions and intellectual functions are affected with general weakness, but in which fits of puerile anger will now and then break out, followed by a long interval of calm apathy: this constitutes folly, *(la demence.)* Lastly, an abolition, more or less complete, of the faculties of the mind and the affections of the heart, is designated by the term *idiotism.*—Those are the four particular species of derangement indicated in a generic manner by the title *mental alienation.*

It is not very uncommon to see one species of alienation, of whatever kind, become transformed into another, and thus furnish examples of a sort of reciprocal affinity. Maniacal delirium may be more or less prolonged, and terminate in a permanent cure, if the mode of treatment be judiciously directed: at other times it may, by the abuse of debilitating measures, or original weakness, become converted into a state of folly. The exclusive delirium of melancholics is not less subject, in some cases, to become general, with turbulent and maniacal agitation; but it still oftener, when it does not give way to the means employed for its relief, becomes habitual, and ends in incurable folly. On the other hand, an accidental state of folly, appearing in youth, and from the abuse of debilitating agents, may be cured by a temporary attack of maniacal agitation. A violent and sudden mental emotion may convert hypochondriasis, or hysteria, into a sort of idiotism; while, in other instances, the most violent mania will be the result. It appears that original idiotism generally arises from organic defect in the brain, and is not liable to any sort of transformation, being as durable as the physical cause on which it depends.

Mental alienation, generally considered, may evince different derangements of physical sensibility; and it is thence that mania is, for the most part, characterized by extreme agitation, burning heat, great increase of muscular force, and, at irregular intervals, an insatiable voracity for food. The melancholic, on the contrary, plunged in deep reverie, seeks inactivity and repose, and will support, without complaining, hunger, thirst, and every possible hardship of life. So many physical and moral causes will be productive of folly at all ages and in all constitutions, that it will, in some instances, be connected with the most energetic activity; in others, with the most dull slothfulness, and the utmost degree of muscular debility. Idiotism seems to deprive its victims of all the most precious gifts of human nature; but, what extraordinary energy of the functions of digestion and generation does it sometimes confer!

Should not the different lesions of the faculties of the mind, in alienation, be relatively determined in particular instances, with the state of health? But how are we to decide on the number and character of the primitive functions, through all the variety of energy and debility, of rectitude and falseness of judgment, of intelligence and ignorance, of which the human species offers examples, independently of disease? But, without entering in this place on discussions on ideology, we may ask, can it be denied that man receives his sensations from impressions on certain organs, and that he continues to retain the conception of the external objects which have produced them? Can the power of directing his attention according to his will, and of associating or rearranging different series of ideas, be denied him? Is the memory not a primitive function of the understanding; and is it not adapted to furnish materials for the imagination, which, by variously combining them together, may from them create new ones, and thence form a vast whole? It now remains for us to give our readers some idea of the different lesions of the understanding, according to the different species of mental alienation.

The greater number of maniacs suffer no absolute change of the senses of sight, hearing, or touch; but the functions of some of those organs of sense may be perverted, and give rise to involuntary errors: the illusion may perhaps even be carried so far, that present objects may not be perceived, and fantastical images taken for real objects. I was once consulted by an insane person, who could not be made to doubt of the truth of the metamorphoses of

Ovid, and who rejected the approaches of her husband with indignation, under the pretence that a stranger might have assumed his external form. Another maniac believed himself to have been transported into paradise, and to converse familiarly with angels. Some think they are surrounded by serpents and other objects of terror. These aberrations of the functions of the organs of sense have a still greater and more settled influence on the understanding of some melancholics, who believe themselves to be the victims of imaginary persecutions; some of whom see poisons, in the form of dust, escape from the boards of their chamber; others think their nerves are acted on by means of a magnetic or electric emanation, and suppose they can distinguish the rays of the effluvium. In many cases of folly, the perception of objects, although not false, is so feeble and vacillating, that hardly any trace of them is left; and often none whatever in idiotism.

In many cases of mania, we observe a sort of continual garrulity, which announces a rapid succession of tumultuous and incoherent ideas, when the attention cannot be fixed on any object, and in which, indeed, this function of the understanding appears to be entirely abolished; but often, in less violent cases, amongst the eccentric wanderings of maniacs, we may fix their attention on some new object, and relieve them from the continual mobility of ideas with which they have been led away. I have related, in my work on *Mental Alienation*, the case of an insane person who, in the midst of a paroxysm of delirium, became calm for some time, and wrote a very sensible and appropriate letter to me: a temporary effect, produced by a circumstance which had confined his attention. This function is exerted in too constant and powerful a manner in melancholy, since it is exclusively confined to a particular series of objects, that appear entirely to absorb it, and by that means to obtrude the greatest obstacle to the means of relief. This difficulty is still augmented, if to these unpleasant circumstances are added the extreme depression and despair which sometimes distinguish hypochondriasis. It is useless to speak of attention relative to folly or idiotism, since, in this species of alienation, it is either very imperfect or absolutely abolished.

To conceive with accuracy, and faithfully to represent, a physical object, after it has ceased to impress itself on the senses, is the attribute of a clear understanding: to retrace it wholly and in detail in methodical order, and paint it in appropriate colours, is the fruit of rare and cultivated talents. Some maniacs, carried away by a rapid succession of ideas, glance superficially on every object which strikes their senses, but their conception of them is very incomplete; at other times, it is disfigured by preconceived or chimerical ideas: but, in a great number of cases, the maniac seizes with clearness and precision the distinctive characters of objects, representing them to himself with force and vivacity. Melancholy is, in general, conjoined with great strength of perception, and the most energetic passions: thus the subjects of predisposition to this species of delirium are often well adapted for the cultivation of the arts and sciences.

Memory, like all the other primitive functions of the understanding, appears to be suspended during the violence of certain paroxysms of mania, and it is only on their decline that it resumes its powers. The victim of this species of derangement often possesses neither remembrance of his delirium nor of his extravagant conduct; and he can hardly be persuaded that he has remained so long in a state of confinement as the registers of the hospital attest. We may, however, cite contrary examples; for some insane persons preserve the remembrance of all that passed during the most turbulent state of their delirium, and, in their lucid intervals, express the most poignant regret for the impropriety of their actions: they will often avoid the presence of those who have seen them in that state, as if they might reproach them for the involuntary improprieties of their conduct towards them. Sometimes, also, ideas which had long previously occurred to their mind will be renewed with extreme vivacity, and seem to efface or render void the impressions of present objects. In melancholy, gloomy suspicions, and the ideas which give rise to inveterate hate, leave the most profound traits in the memory, and appear even to render themselves permanent in certain incurable cases. Folly and idiotism most frequently produce the most perfect obliteration of memory, or rather only suffer a sentiment of gloomy stupor to remain.

The different primitive functions of the understanding are then, more or less destroyed, perverted or seriously affected, or only one among them deranged, in the different species of alienation; their historical description may be thus facilitated, in confining it to exterior unequivocal signs: on the other hand, it is by placing in opposition to the state of health the different species of derangement observed in a large hospital, and conducting our researches with strictness and precision, that we may be enabled to fill up the vacant spaces which yet remain in ideology, illustrate some difficult points which have not hitherto been explained, and contribute to the steady progress of this yet wavering branch of human knowledge.

A natural reflection arises from the general consideration of the different modes of treatment that have been adopted by the ancients and moderns: it is, that the idea of a pretended specific, at least if it be not founded on an extensive series of well-determined facts, leads us on this subject, as in many others, to a blind empiricism and a continual vacillation of opinions. The ancients, although accurate observers, drew general conclusions from a too-confined a number of particular facts; and, referring the seat of the disease to the digestive organs, they believed the only means to be employed, in all cases, were drastic purgatives. The greater number of moderns, on the contrary, have only regarded the alienation as a consequence of too great an afflux of blood to the head, and have merely advised a repetition of frequent blood-letting, and the application of cold water to the head. Other physicians have considered it as a purely nervous or spasmodic disease, and have had recourse to anodynes and sedatives exclusively. But a judicious application of the medical art in a large assemblage of patients of this class, shews us that these are only modes of treatment relating to the different circumstances which have produced or encouraged the malady, and the individual constitution, age, &c. of the patient.

The manner of directing an hospital of lunatics, with respect to the rules of hygïene, is subjected to the general rules which are common to it with that for the general assemblage of infirmities; but there are some indications which are peculiar to it, naturally arising from its particular application: as, in the first place, a spacious local residence divided into numerous apartments; a pure and salubrious air, that may be made to circulate freely through all the different cells; and sometimes a necessity for disinfecting them by means of fumigations of muriatic acid: the impression of cold air is dangerous in the decline of a paroxysm; the use of mucilaginous decoctions acidulated, or emulsions; during the state of agitation and violence of mania, or on its decline, the exhibition of gentle laxative medicines, alternately with a light bitter tonic infusion; in general, a substantial nourishment, but principally composed of vegetable substances; autumn and summer fruits are not less useful for maniacs than for melancholics; not to condemn to inaction any but the violent or dangerous maniac, and to permit him to use his limbs as soon as he is no longer extravagant, or the violence of the paroxysm has subsided. Does he become convalescent?—to apply him to some sort of occupation adapted to his inclination; to occupy women with needle-work, and the men with some mechanic labour, or gymnastic or rural exercise; and, in some cases, to favour certain natural secretions or excretions, according to the particular cause which has determined the production of the malady, or to produce some preternatural evacuation. The moral and physical treatment will also require to be modified according to the different periods of mania.

In the first period,—is the patient in a state of extreme agitation?—let him be confined in an obscure chamber, to avoid the impression of light, and all objects tending to produce excitement; and to indulge his inclination for cooling and acidulated drinks. He often, then, experiences at intervals a great voracity, when an abundant quantity of nourishment should be allowed him. When the violence of the paroxysm diminishes, and it will be no longer dangerous to himself or others, he may have the liberty to use exercise in a confined space, his motions being in some degree restrained by a strait-waistcoat. This is, then, merely a sort of *medecine expectante*, aided by a mild diet proportioned to the appetite; a careful superintendance of his actions, regularity of attendance on him, and a sort of harmony between all the objects conducive to health. I revere the ancients, I admire their supreme sagacity in the art of observation; but I may also be allowed to be guided by the results of more than twenty years' experience on extensive assemblages of patients, which has led me to confine to certain particular cases the use of emeto-cathartics, hellebore, gratiola, sternutatories, repeated blood-letting, sudden immersion in cold water, so blindly and servilely adopted, without restriction, by some modern physicians.

On the decline of the disease, greater liberty of movements may be gradually permitted, and patients may be drawn from a state of fury and violence, by having recourse, at intervals, to a variety of gentle measures,—as, tepid baths two or three times a-week; the occasional use of laxative and acidulated drinks; gentle dashing of cold water on the head, and this only towards the end of the time at which they remain in the bath, and for a few minutes; all the resources of moral regimen; testimonies of kindness and affection; condescension to all his little whims, and a careful evasion of his indiscreet demands; acts of violence, nor even offensive commands, should never be employed, but a regular, firm, imposing style of conduct, whenever the patient assumes an authoritative tone or becomes unruly.

The general views which have been displayed of the mode of treatment for lunatics, may be modified by the particular circumstances of the determining cause, the individual constitution, the mode of life, and the various complications of the malady. If it have been preceded by the repulsion of some cutaneous disease, or of gout, these disorders should be recalled to their original seat by irritative frictions, blisters, and setons, joining with these external means, antimonials, sudorifics, sulphureous waters, &c. Should the disorder appear during pregnancy, we must patiently wait for the termination of this state. Does it take place after childbirth? —constant experience teaches us the advantage to be derived from evacuants in the first instance, followed by the application of a vesicatory to the neck. A plethoric state of the system, or the suppression of some hæmorrhage, may render bleeding necessary, but not a frequent repetition of it, which may plunge the patient into a state of incurable folly; but local abstraction of blood by leeches applied to the inferior extremities, sometimes to the anus, to the vulva, or to the head, according as a suppression of an hæmorrhoidal evacuation, a periodical flux, or the preludes of cerebral congestion, may have been remarked.

A lunatic subject to spasms, should be treated in some degree with anodynes and sedatives: a state of atony and stupor demands, on the contrary, the use of stimulants and tonics. We have previous indications for the mode of treatment when insanity is determined by a wound of the head, disappointed or rejected affections, a forced state of celibacy, difficult dentition, habitual insomnicum, a state of extreme distress, &c. Is it not curious to see a physician seek his means of cure, in these cases, in pharmacy?—Pinel.

### *On the Medical Measures employed by Brute Animals.*

The following interesting reflections on the above-mentioned subject are taken from the *Journal de Pharmacie* (June 1818): they are by the enlightened and philosophic Virey.

Hippocrates admitted the existence of an intelligent principle, which governed brute animals as well as men (says Galen, l. i. *de Utilit. part.*, c. 3); and this opinion seems to have generally prevailed during the middle ages. (Averroes, in 7. *Physic;* Albertus Magnus, lib. iii. *de Anima;* Philoponus, *ad text.* 155; Laurentius Valla, *Dialect.* c. 9; Cardan, Hieron. Majius, *de Exust Mundi*, l. ii. c. 7, &c.)

Among the moderns, Cudworth explained this principle under the term *Plastic Nature;* but the writer that appears to us to have best developed the history of this astonishing faculty is Hermann Samuel Reimar, professor of philosophy at Hamburgh: (see his *Observations on Natural History*, &c. in German; Hamb. 1801, in 8vo.) Darwin, who wrote much on instinct in his *Zoonomia*, did not properly distinguish it from intelligence, any more than the greater number of modern writers on this subject. The celebrated Emen. Kant, however, opened the way to research, by recognising primitive and innate faculties of the mind; and particularly Cabanis, who, although a disciple of Locke, acknowledged those interior sensations which perceptibly sway our intelligence even during sleep.

It is much to be wished that we could witness the researches of modern medicine more directed to those important indications of instinct in the infant, and particularly in sick men and brute animals. It is by observation of the latter, that we should penetrate most deeply into the knowledge of our spontaneous conservative efforts. That beasts have been our first physicians, we cannot doubt, even though it were not attested by the history of medicine. *Syrmaïsm,* or purgation of the intestinal canal universally, was pointed out to the Egyptians, by the vomiting dogs' produce by means of dog's-grass, says Ælian (*Hist. Anim.* lib. v. c. 46). This observant people learnt the use of blood-letting from the hippopotamus, (Cicero, *de Natur. Deor.* l. ii.) The ibis taught them to employ clysters, says Galen (*de Venæsect.* c. i.), and Plutarch (*de Brutor. Solert.* and *de Isid. et Osir.*), and Pliny (*Hist. Nat.* l. viii. c. 27.) The good effects of saliva in the cicatrization of ulcers, has been shown by dogs, who lick their wounds. (Ælian, lib. viii. c. 9, Joh. Schmid, *Diss. de Brutis Hominum Doctoribus*, Lips. 1684, and Paul Boccone, *de Solertia Brutorum in se ipsis Curandis*, and Mauget, *Bibl. Med.* tom. i. part i. art. 25.) In low marshy grounds, beasts afflicted with dropsy swallow ferruginous earths, just as chlorotic girls and pregnant women do, from *instinct.* The internal voice of the animal organization is so manifest in many diseases, in brute animals particularly, that in this respect even bears may furnish us with more instruction than men.

A sort of instinctive mechanism makes a plant, bent from its natural course, tend to regain its original direction, and precisely the same direction, whether erect or creeping, to which it was assigned by nature. We see it turn its foliage to seek the light, and close its flowers during the night. A similar instinctive mechanism determines an animal to the exercise of every action to which it was predisposed, even though it may be deprived of the organs with which it was to execute them. Thus, although the horns may have been cut from a bull, the claws from a cat, or the sting from a scorpion, these animals do not the less act as if they possessed their arms; they preserve them in their mind. A similar cause makes the larynx and glottis become closed when we swallow, and induces coughing when any thing penetrates within that organ. In the same manner, the stomach rejects poison with horror, and the nasal membrane the snuff which irritates it. Our natural passions, as our appetites, are all instinctive directions, with which it is so essential that a physician should be acquainted. *Quod autem membra et partes sigillatim in omni motu et omni appetitu concurrant, et quo pacto singulæ moveantur, scire non facile est; immò quàm difficillimum, at medicis valdè necessarium.* —Hier. Fracastorius, *de Intellectione*, l. ii. p. 136, &c.

No one more carefully consulted instinct than Sydenham; and it is to his precise direction that we are indebted for the abolition of the pernicious mode of treating variola by heating medicines, diaphoretics, and confinement to a hot bed, which made this inflammatory disorder in the highest degree dangerous. We should doubtless escape many diseases if, following the good instinctive directions that nature points out even to brute animals, we did not act in opposition to the voice of health, which speaks to us from within when we are inclined to consult it. (See *le Médecin de soi-même*, La Haye, 1699; a work of John Deavaux; also Hilscher, *Program.* 2, *de sensu corporis, sanitatis, conservandæ, et reintegrandæ consiliario*, Jena, 1729; et Maizien *Dissert. de Instinctu*, Halæ, 1796.)

Whence did we derive our knowledge of the medicinal properties of plants?—it was from animals, as Plutarch affirms: ὃτι ἀλητὴς ἐστι τουτων διδασκαλον εἶδαι τὴν φύσιν. The deer and wild goats of Crete first pointed out the use of dittany and the vulnerary herbs, according to Cicero, Virgil, and other ancient authors: but, admitting that there is some degree of gratuitous supposition in saying with some of them, that the swallow clears its sight by rubbing the cornea with the juice of chelidony; and that serpents have shewn the use of fennel (Ælian, *Hist. Anim.* lib. iv. c. 9); and the toad that of the narrow-leaved plantain, (Van Helmont, *Tumul. pestis*, &c.); yet it is very probable that nature, far from abandoning its weakest creatures, has furnished them with the means of relieving them from accidental evils. When we see the smallest insects, immediately on escaping from the egg, without a guide on the earth, discover precisely the plant which is best adapted for them, the nectar hid at the bottom of a flower; and, if they do not find the vegetable naturally destined for them, to feed on other vegetables of the same genus, or even the same family, like an experienced botanist,—(such is the case with many insects of America, transported into Europe with merchandise,)—we may be induced to believe that divers animals have dictated to us empirical medicine. It is a general tradition in India, according to Kempfer, Garcias *ab orto*, and other voyagers, that the mangouste secures itself from the venom of the serpent *naja*, by means of the root *ophiorrhiza mungos*, L. It has been said that weasels, in like manner, preserve themselves from the venom of asps by means of *rue*, and the crane with *origanum*; that wild boars cure their wounds with ivy; that the bear, in the spring, restores his appetite either with *arum*, which purges him, or by devouring *ants*; that deer taught us to eat artichokes, and other species of *cynara*, &c. It is certain that cats and other carnivorous animals change their diet, and drink copiously of water, when they are sick. HEDMANN has seen the apes of America, and the supajous of Guyana, in the forests, apply certain astringent and aromatic leaves, chewed, on the wounds they received from the arrows of the savages, and stop the flow of blood with the gum of trees, &c.

Have not animals ingenious provisions, and presentiments of the changes of temperature of the atmosphere, the approach of storms, and even of earthquakes, and other natural phenomena, which men certainly do not possess in an equal degree.) AELIAN, *Anim.* l. vi. c. 16. Don ULLOA also states, that at Quito dogs have a presentiment of earthquakes, &c.) How do water-fowl prognosticate the approach of rain, and particularly mallards, swans, and all the white-feathered *palmapedes*. (AELIAN, l. vii. c. 7)? Certainly the emigration of birds has fixed periods; and a thousand other remarkable circumstances, embellished by VIRGIL with all the charms of poetry, announce that the observation of *auspices* and *aruspices*, among the ancients, were not solely objects of superstition. Leeches, water-frogs, and a great number of other animals, which, being very sensible to the electric state of the atmosphere, may serve as aquatic barometers, have been often consulted with advantage, not only by peasants, but by enlightened philosophers.—See Th. Hoffmann, *de Præsagiis Tempestatum Naturalibus*, Basil, 1781; Just. Cellarii, *Diss. de Penetrabili efficacia Effluviorum in efficiendis Animalium Corporibus*, Resp. Behrens, Helmst. 1684; Rud. Chr. Wagner, *Meteorologia Animal. Brutor.* Resp. Wahrendorf, Helmst. 1702; Joh. Chr. Ortlob, *de Brutor. Præsag. Natural.* Lips. 1702; Geo. Loffhagen, *de Vaticiniis Brutor.*; in the *nov. Litt. Maris Baltici*, 1703, p. 255; and Sam. Œdmann, *Calendarium Faunæ*; in the *Neuen Abhandl. der K. Schwed.* akad. 3, b. p. 148, &c.

---

THERE are few medicinal substances, on the qualities of which greater diversity of opinion prevails among physicians, than the *Arbutus Uva Ursi.* Like digitalis, cicuta, and several other powerful remedies, it has fallen into general disrepute from the unreasonable expectations excited respecting its efficacy in diseases to which it is not applicable. A conviction, from experience, of the useful properties of Uva Ursi in some distressing diseases, and a desire to call the attention of medical practitioners to this remedy, have induced us to present to our readers an account of its botanical characters and medical properties. We are aware that much has been written respecting it by Oeder, Woodville, Smith, Pursh, Lobel, Parkinson, De Haen, Murray, Fothergill, Ferriar, Heberben, Davie, Schoepf, Bourne, Mitchell, and others; but we have considered that a general deduction from the opinions of these authors may neither be devoid of interest nor of utility. The following account is principally taken from an erudite and scientific work on Medical Botany, in the course of publication, by Dr. Bigelow, of Boston in the United States; two parts of which have already appeared.

Few shrubs are more extensively diffused throughout the northern hemisphere, both in the old and new continents, than this trailing evergreen. It abounds in Sweden, Lapland, and Iceland, and extends southerly to the shores of the Mediterranean. In Siberia it is also found, and is represented as abundant on the banks of the Wolga. In North America, it grows from Hudson's Bay as far south, at least, as the central parts of the United States. It occupies the most barren places, such as gravelly hills, and dry sandy woods, and covers the ground with beds of considerable extent.

The family of plants bearing the name of Arbutus have for their distinctive marks a *five-parted calyx*, an *ovate corolla*, *pellucid at the base*, and *a superior five-celled berry*. They are closely connected to the Vaccinia, or whortleberries, from which they differ principally in the situation of the berry, which in the arbutus grows above the calyx, and in the vaccinium below it. Both these genera, at least the American species, properly belong to the class *Decandria*, and order *Monogynia*. The Linnæan natural order is *Bicornes*; Jussieu has them among his *Ericae*.

The species Uva Ursi, bear's-grape or bear-berry, is known from the rest by its procumbent stem and entire leaves. It trails upon the ground, putting out roots from the principal stems, and tending upward with the young shoots only. The cuticle is deciduous, and peels off from the old stems. Leaves scattered, obovate, acute at base, attached by short petioles, coriaceous, evergreen, glabrous, shining above, paler beneath, entire, the margin rounded but scarcely reflexed, and in the young ones pubescent. Flowers in a short cluster on the ends of the branches. Peduncles reflexed, furnished at the base with a short acute bracte underneath, and two minute ones at the sides. Calyx of five rounded segments, of a reddish colour, and persistent. Corolla ovate or urceolate, white with a reddish tinge, transparent at base, contracted at the mouth, hairy inside, with five short reflexed segments. Stamens inserted at the base of the corolla with hairy filaments, and anthers with two horns and two pores in each. Germ round, style straight, longer than the stamens, stigma simple. Nectary a black indented ring, situated below the germ, and remaining until the fruit is ripe. Berries globular, depressed, of a deep red approaching scarlet, containing an insipid mealy pulp, and about five seeds, which in the American plant cohere strongly together, so as to appear like the nucleus of a drupe.

The uva ursi was probably known to the ancients, as it grows in all the southern parts of Europe. Clusius thinks it was the *αρκτου σταφυλη* of Galen, celebrated by him as a remedy in hæmoptysis, and described as follows:—"Uva ursi in Ponto nascitur, planta humilis et fruticosa, folio Memæcyli, fructum ferens rubrum, rotundum, gustu austerum." But the brief and imperfect descriptions of the ancients were productive of little else than uncertainty in botany.

In modern times, the uva ursi was brought into notice about the middle of the eighteenth century, by De Haen, as an efficient remedy in nephritic, and even in calculous, cases. It had been previously in use for these complaints in Spain, at Naples, and Montpellier; and, as a general astringent, at a much earlier period. Its reputation was still further augmented by subsequent dissertations published upon its properties; and different sets of experiments were instituted to ascertain if it were not actually capable of dissolving the stone in the bladder.

The attention of many medical writers has been called to the properties of this medicine, and their reports as to its success are extremely various. Among those who think highly of it are De Haen, Professor Murray, and Dr. Ferriar; while Sauvages, Haller, Donald, Monro, and Fothergill, have an unfavourable opinion of it. Dr. Cullen adopts the opinion of De Heucher, that the symptoms of calculus generally are susceptible of relief from astringents, and believes that, on this principle, the uva ursi is capable of mitigating complaints arising from that source.

In America, the uva ursi has obtained the good opinion of practitioners of medicine in repeated instances. Professor Wistar of Philadelphia, as cited by Dr. Mitchell, has in several cases found symptoms like those of urinary calculus completely removed by this medicine. The late Professor Barton found the plant of much service in his own case of nephritic paroxysms, alternating with gout in the feet.

From the various testimonies which have been given respecting the properties of this medicine, although we cannot suppose it to possess any real lithontriptic power, yet it appears that it undoubtedly proves a palliative for calculous symptoms in many cases.

Dr. Bigelow states, that he has repeatedly watched its effects in paroxysms of nephritis, brought on by gravelly concretions, and is inclined to believe that it tends to allay sensibility in these cases, and to hasten the relief of the symptoms. It ought generally to be preceded by evacuations, and may be advantageously accompanied with opium. In cases of dysury, arising from a variety of causes, he has given the decoction of this plant with very satisfactory success in repeated instances.

The other diseases in which this plant have been recommended are, catarrhus vesicæ, leucorrhœa, and gonorrhœa.

Some years ago the uva ursi was recommended as a remedy in pulmonary consumption, by Dr. Bourne of Oxford, and by other writers in several periodical works. It was stated to have a very sensible effect in diminishing hectic fever, and abating the frequency of the pulse dependent on it. We do not find, however, that subsequent experience has justified the expectations formed of it in this disease.

In Dr. Mitchell's experiments on the pulse with this medicine, it appears that the pulsations were sometimes, not always, slightly increased after taking it; but that, in every case, they soon sunk below the natural standard, and remained so for some time.

If uva ursi shall be found to contain prussic acid, as we suspect that and all the species of drupaceous plants do, its beneficial qualities may then become better known and more generally acknowledged.

